# Orthopædics in General Practice.—II

**Published:** 1908-12-19

**Authors:** 


### ORTHOPÆDICS.

### ORTHOPÆDICS IN GENERAL PRACTICE.—II.

The general practitioner cannot very well add to his multifarious duties by attempting to rival the orthopædic specialist; he lacks the time and the means for ensuring success, not to speak of the manipulative dexterity and skill which are only attained by constant and very varied practice. There has been a tendency, therefore, on his part to regard deformities as the worst thing he has to deal with. The flat-foot and knock-knee he regards as beyond reach of his remedy; they are things to be sent to the operating surgeon. Similarly, "only more so," he looks upon paralytic contractions and the results of anterior poliomyelitis. We are not reflecting upon his methods or maligning him when we say that the general practitioner treats cases of infantile paralysis as if they were hopeless—somewhat on the same lines as Percival Pott treated his carcinoma of the mammæ, "which he had never seen cured," or his great pupil Richter, of Göttingen, necrosis of a fore-arm bone, which he looked upon as a self-limiting lesion in which Nature cured or killed, in spite of the surgeon's care. Still more unfortunately, there is a tendency to do something in all these cases of paralytic deformities. The patient, or his friends, like to see the doctor exert himself, and if there is a great truth which the profession as a whole has not yet fully mastered, it is that nothing is more foolish than "active uselessness," as Paget phrased it. To most of us the excellent disquisition on the folly of interfering where interference can do no good, and on the wisdom of logically criticising lines of treatment when they have been apparently successful, will be familiar; to those who are unacquainted with it we whole-heartedly commend the classical essay "On the Treatment of Carbuncles." The temptation to be "actively useless" is one that we are all exposed to, and it is nowhere so pernicious in its results as in orthopædic cases. Many a fair case is reduced to an operating case by neglect of preliminary treatment or by bad preliminary treatment. In infantile paralysis cases, for example, the child is irritated and worried by futile electrical applications and strenuous massage; in flat-feet cases, again, the trouble is often made worse by ill-advised "exercises," which are supposed to strengthen the arch, but which really only throw a greater strain on the weakened ligaments that form the string of the bow of the foot.

In all such conditions the usefulness of the general practitioner can be, and should be, great. He sees the case first, and, if he knows the condition, properly diagnoses it, and is familiar with its clinical history, he is in the best position for prognosing as to its future. In orthopædic cases the personal factor is as important as in the usual run of medical or surgical cases. This is a fact that is often lost sight of. The lesion is so obvious usually that its prognosis appears to depend largely on local conditions, but in most instances this is far from being the case. A slight joint injury, such as would lead to no untoward results in a healthy young adult, may become the source of much trouble in a patient who suffers from tabes. Again, a sprained ankle in a healthy patient is a comparatively easy lesion to treat; in one who suffers from gonorrhœa it may become a much more serious matter and lead to a bad flat-foot. The family practitioner, who knows the constitution and habits of his patients, is therefore sometimes in a much better position to prophesy than is the orthopædic specialist who has to rely solely on the story of the patient or the latter's friends.

It is usually held that in private practice orthopædic work is unsatisfactory and difficult, owing to the fact that properly to treat many cases demands complicated and expensive apparatus, much time and trouble, and a large amount of special skill. This is far from being the case. In some instances apparatus is absolutely indispensable, but the expertness and experience of the orthopædist are nowhere shown so vividly as in his employment of them. The best men use the simplest and cheapest apparatus, such as can be turned out by any reliable instrument maker at a moderate price. What ensures their success is that in no case is such apparatus fitted or allowed to be used unless it is absolutely necessary and has been carefully inspected and personally approved of by the surgeon. In many ordinary cases, as we will attempt to show in ensuing articles, the practitioner can do all he wishes to do with the help of such simple accessories as are to be found in every private surgery—scissors, card-board, cork, and bandages. In others plaster and bandages and special glue preparations, simple to make and comparatively inexpensive, are required, and a few pieces of special apparatus which are easily obtained and which, being cheap and useful for a variety of purposes, are handy things to stock in the private surgery. The objection that attention to orthopædic cases entails a great deal of time and skill is true, but the time and trouble spent in treating a case of bad flat-foot will be amply repaid in many ways. We do not say that the practitioner will be able to deal with every case that comes to him. Some there are the treatment of which is justifiable only in a hospital and by specialists of great operative skill, but these are not the cases that will worry the general practitioner so much as the ordinary and usually simple cases of common sprains and deformities. With these latter we hope to deal practically in the following articles.

